# Induction and Maturation of Hepatocyte-Like Cells *In Vitro*: Focus on Technological Advances and Challenges

**DOI:** 10.3389/fcell.2021.765980

**Published:** 2021-11-26

**Authors:** Ye Xie, Jia Yao, Weilin Jin, Longfei Ren, Xun Li

**Affiliations:** ^1^ The First Clinical Medical College, Lanzhou University, Lanzhou, China; ^2^ Key Laboratory of Biotherapy and Regenerative Medicine of Gansu Province, Lanzhou, China; ^3^ Institute of Cancer Neuroscience, The First Hospital of Lanzhou University, Lanzhou, China; ^4^ The Medical Frontier Innovation Research Center, The First Hospital of Lanzhou University, Lanzhou, China; ^5^ The Department of General Surgery, The First Hospital of Lanzhou University, Lanzhou, China; ^6^ Hepatopancreatobiliary Surgery Institute of Gansu Province, Lanzhou, China

**Keywords:** hepatocyte-like cells, hepatocyte induction, chemical approach, culture system, genetic manipulation

## Abstract

Limited by the poor proliferation and restricted sources of adult hepatocytes, there is an urgent need to find substitutes for proliferation and cultivation of mature hepatocytes *in vitro* for use in disease treatment, drug approval, and toxicity testing. Hepatocyte-like cells (HLCs), which originate from undifferentiated stem cells or modified adult cells, are considered good candidates because of their advantages in terms of cell source and *in vitro* expansion ability. However, the majority of induced HLCs are in an immature state, and their degree of differentiation is heterogeneous, diminishing their usability in basic research and limiting their clinical application. Therefore, various methods have been developed to promote the maturation of HLCs, including chemical approaches, alteration of cell culture systems, and genetic manipulation, to meet the needs of *in vivo* transplantation and *in vitro* model establishment. This review proposes different cell types for the induction of HLCs, and provide a comprehensive overview of various techniques to promote the generation and maturation of HLCs *in vitro*.

## 1 Introduction

Liver transplantation is the only therapeutic modality for curing end-stage liver disease. However, the chronic shortage of donors has compelled researchers to develop alternative treatments. Clinical studies have demonstrated that transplanted hepatocytes can relieve patient symptoms, prolong their survival (Hansel et al., 2014), and provide a “bridge” therapy until patients are matched with an appropriate liver for transplantation (Nguyen et al., 2020). However, there are problems associated with human hepatocyte transplantation. First, human primary hepatocytes have higher cell quality requirements, and isolated hepatocytes lose their functionality after prolonged periods of culture *in vitro.* In addition, long-term oral immunosuppressive drugs are needed after allogeneic hepatocyte transplantation which has arisen adverse effect and given negative impact of patient’s life quality. (Zeilinger et al., 2016; Miki, 2019; Ruoss et al., 2020).

In theory, undifferentiated stem cells can be induced into hepatocytes along the development track of hepatocytes under external intervention. The final induced cells were shown to adopt the phenotypes of hepatocytes, express hepatocyte-specific genes, perform glycogen storage and albumin synthesis functions. However, when compared with human hepatocytes (HHs), most of these cells express higher level of alpha-fetoprotein (AFP) and, perform insufficient detoxification functions, so called hepatocyte-like cells (HLCs). (Bell et al., 2017; Roy-Chowdhury et al., 2017; Cotovio and Fernandes, 2020). Nevertheless, even with this immature state, HLCs show an ideal effect in treating animal models of liver diseases and, are used for generating *in vitro* organoid models for predicting the hepatotoxicity of new drug (Corbett and Duncan, 2019). Unfortunately, immature phenotypes and the inconsistent differentiation of HLCs in the same batch, especially those derived from stem cells, pose a risk of tumorigenesis after transplantation into humans (Xu et al., 2018). All of these obstacles block the transformation of HLCs as an alternative to HHs in clinical applications, and greatly discount the authenticity of drug prediction results in some basic experiments, because HLCs cannot fully express the function of mature hepatocytes.

**FIGURE 1:**
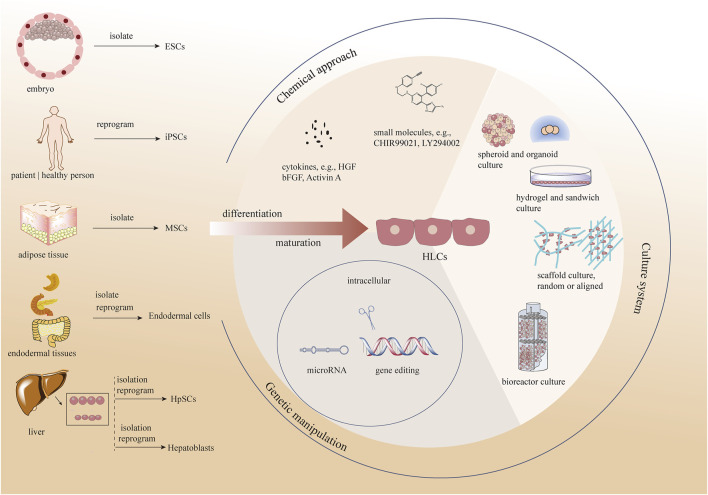
Different methods promoting the maturation of HLCs *in vitro* as well as various cell sources for HLCs formation. ESCs, embryonic stem cells; iPCSs, included pluripotent stem cells, MSCs, Mesenchymal stem cells; HpSCs, hepatic stem cells; HGF, hepatocyte growth factor; bFGF, basic-fibroblast growth factor.

Thus, the question arises as to how HLCs can be generated with similarities to HHs both for *ex vivo* use and towards eventual clinical programs. Researchers have developed several methods to promote hepatocyte maturation by attempting to simulate hepatocytes *in vivo* for liver progenitors to induce mature and stable HLCs *in vitro* (Berger et al., 2015; Touboul et al., 2016). Actually, these methods can be divided into three types, chemical approaches, changing the culture system, and genetic manipulation. In this review, we discuss various cell sources for HLCs formation and methods promoting the maturation of HLCs *in vitro* (Figure 1).

## 2 Cell Sources for Generating Hepatocyte-Like Cells *in vitro*

**FIGURE 2:**
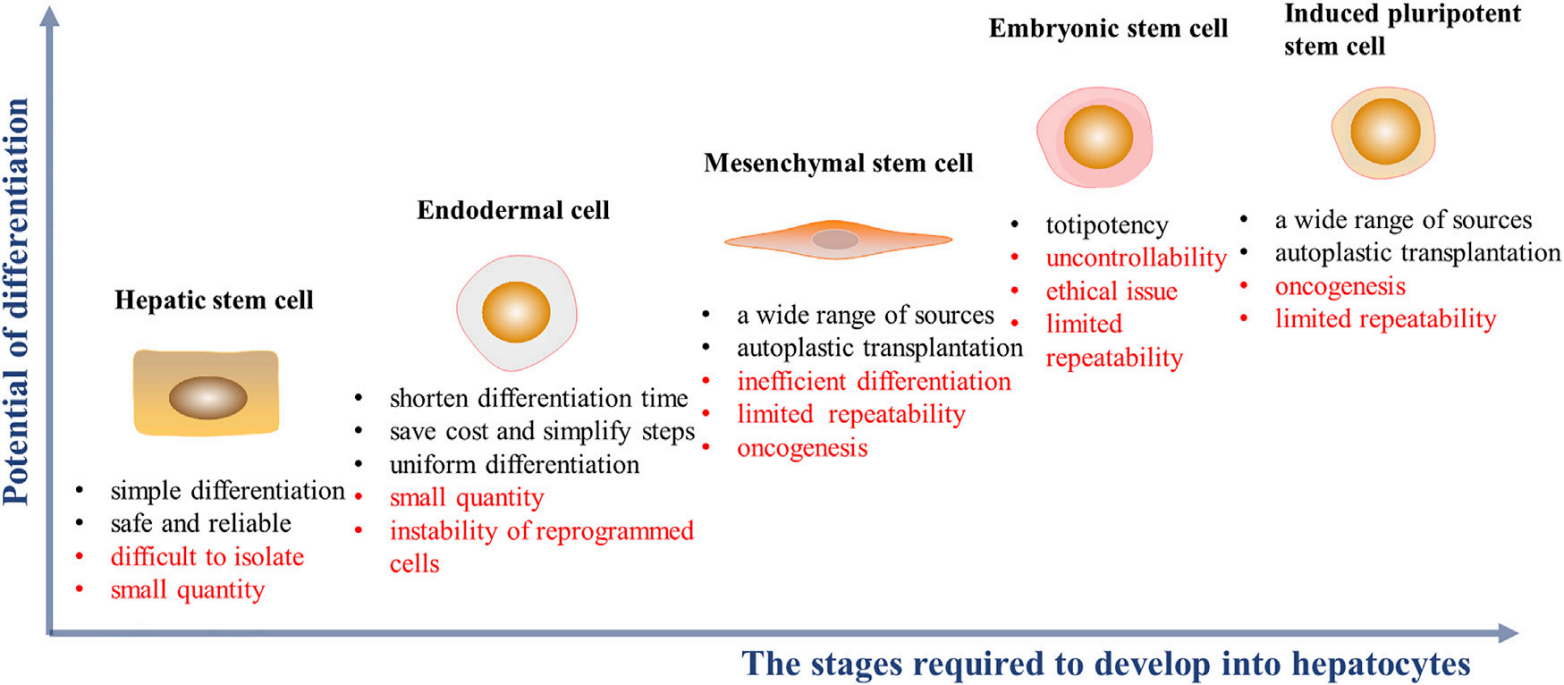
Cell sources of HLCs induction *in vitro* and their advantages (black font) and disadvantages (red font). The abscissa represents the complexity of the stages required during hepatic differentitation. The ordinate represents the potential of cell differentiation into other cell types.

HHs are considered the “gold standard” for functional cells used for drug screening and for cell transplantation. It is noteworthy that neonatal hepatocytes, compared with adult hepatocytes, have higher viability with better treatment outcomes in clinical settings, even after cellular cryopreservation (Tolosa et al., 2014; Lee et al., 2018). However, owing to a chronic, global shortage of donors, and ethical issues, alternative cell sources are needed (Zeilinger et al., 2016; Ruoss et al., 2020). Studies shows that HLCs can be derived from embryonic stem cells (ESCs), induced pluripotent stem cells (iPSCs), mesenchymal stem cells (MSCs), endodermal cells and hepatic stem/progenitor cells. HLCs, performing some characteristics of hepatocytes, can be a promising alternative of hepatocytes to be tested in some preclinical researches which need to consume sufficient number of cells (Zhou et al., 2017; Wang et al., 2018; Mun et al., 2019) (Figure 2). Some key features of ideal HLCs cell source scientific research and clinical application are sufficient, accessible, and restricted differentiation into hepatic lines with complete phenotype and function in scientific research and clinical application.

### 2.1 Embryonic Stem Cells

Embryonic stem cells (ESCs) feature the pluripotency to differentiate into endoderm, mesoderm and ectoderm. ESC lineages may be restricted to cells with hepatocyte-like features under induction conditions (Mun et al., 2019). ESCs with a comprehensive spectrum are more likely to differentiate into other lineages, which leads to heterogeneous differentiation of HLCs. Before differentiation, if ESCs are transformed into definitive endoderm for narrow-spectrum differentiation, the differentiation efficiency can be improved. However, this may increase the number of steps and duration of differentiation. In addition, it is necessary to provide an appropriate environment for stem cells to support their pluripotency when cultured *in vitro*. Generally, according to the materials of substratum on the dish, the culture methods are divided into feeder-dependent culture (e.g., mouse embryonic fibroblasts and skin fibroblasts) and feeder-free culture (e.g., Matrigel, collagen, human recombinant laminin and its subtypes) (Hoffman and Carpenter, 2005; Dakhore et al., 2018). It has been found that some cytokines and extracellular matrix components secreted by the feeder layer into the culture medium can form a benefit environment for growth of stem cells. For example, basic fibroblast growth factor (bFGF), transforming growth factor-β (TGF-β), and Laminin-511 secreted by fibroblast feeder layer is associated with the cell self-renewal and pluripotency maintenance of human ESCs (Stacey et al., 2006; Hongisto et al., 2012; Lim et al., 2019). Limited by the potential of unidentified pathogens from feeder cells, feeder-free culture represents a greater prospect (Llames et al., 2015). At present, some fully defined commercial media for feeder-free culture, such as E8 and TeSR, optimizes the passage and maintenance of stem cells (Lim et al., 2019). Compared with TeSR medium, the development of E8 medium rejects the animal derived bovine serum albumin and some non-essential additions, simplifying the medium components, maintaining the undifferentiated proliferation of ESCs, and further reducing the culture cost (Ludwig et al., 2006; Chen et al., 2011).

### 2.2 Induced Pluripotent Stem Cells

Induced pluripotent stem cells (iPSCs), which represent a promising source of HLCs, can be reprogrammed from different adult cells (Wang et al., 2016; Roy-Chowdhury et al., 2017). The classic reprogramming technique involves introducing Oct4/Sox2/KLF4/c-MYC genes into candidate cells to reverse cells from a differentiated state to the ground state with the ability to re-differentiate (Takahashi and Yamanaka, 2006). However, the reprogramming efficiency is affected by the expression level of the four transcription factors, and the method poses a potential risk of insertion mutation; furthermore, the continuous expression of *c-MYC* may pose a risk of tumorigenesis *in vivo* (Xu et al., 2018; Haridhasapavalan et al., 2020). Based on efficiency and safety considerations, reprogramming methods have been explored and optimized, such as reducing or replacing the application of c-MYC (Huang et al., 2018; Nakagawa et al., 2008), shifting from genetic integration to integration-free methods, or using small-molecule cocktails for direct reprogramming (Fusaki et al., 2009; Okita et al., 2011; Ma et al., 2017). The culture and induction methods of iPSCs *in vitro* are very similar to those of ESCs. iPSCs are differentiated into HLCs through three stages: endoderm formation, hepatic specification, and maturation (Li et al., 2019). In addition, transcription factor-based reprogramming retains the epigenetic memory of donor cells, which may favor iPSC differentiation along the original tissue and limit the efficiency of differentiation into other lineages, without contributing to performance of the complete phenotype of HLCs induced from liver-derived iPSCs (Kim et al., 2010; Calabrese et al., 2019). The emergence of iPSCs provides a sustainable concept for high-value precision medicine; the use of patient-specific recombinant iPSCs can not only solve the problem of cell source but also avoid various risks related to inhibition and rejection in *in vivo* applications; however, the reprogramming efficiency and culture mode of iPSCs need to be further optimized.

### 2.3 Mesenchymal Stem Cells

Mesenchymal stem cells (MSCs) are a type of non-hematopoietic stem cells that exist in a wide range of tissues such as bone marrow, adipose tissue, menstrual blood and umbilical cord (Raoufil et al., 2015; Farnaz Sani et al., 2016; Xing et al., 2016; Cipriano et al., 2017; Xu et al., 2017). Among them, umbilical cord mesenchymal stem cells are widely studied as the candidate for the treatment of end-stage liver disease and HLCs differentiation (Talaei-Khozani et al., 2015; Varaa et al., 2019). Typically, liver-specific induction and maturation stages are required to obtain HLCs, which are unstable and inefficient due to the need to transition from mesoderm to endoderm (Vojdani et al., 2015; Huang et al., 2017). There is a type of stem cell localized in the liver with a phenotype similar to that of MSCs, can be successfully differentiated into HLCs (Najimi et al., 2007; Lee et al., 2020). Even that being of hepatic origin, these cells are not more mature in hepatic differentiation compared with extrahepatic MSCs (Chinnici et al., 2019). Of course, as an accessible cell source of HLCs *in vitro*, MSCs have the advantages of their low immunogenicity *in vivo*, strong proliferation ability *in vitro*, and unaffected cell vitality and differentiation ability after cryopreservation. However, it is noteworthy that adult stem cell actually accounts for only a small part of the tissue, and the number and proliferation ability of MSCs will decrease along with donor age.

### 2.4 Endodermal Cells

Organs from endodermal origins, including the gallbladder, pancreas and intestine, which are of the same germ layer origins as the liver, also contain endodermal stem cells (Carpino et al., 2014; Lanzoni et al., 2016). These cells can differentiate into HLCs with a shorter differentiation path. Isolation of tissue-derived endodermal stem cells cost far less than that the pluripotent stem cell-derived. The issue with the tissue-derived endodermal stem cells is their *ex vivo* expansion limitation due to the underdeveloped expansion condition. Therefore, pluripotent stem cells are recombined into stable and expandable endodermal progenitor cells as a new cell-type source of HLCs *in vitro* (Cheng et al., 2013; Sambathkumar et al., 2018). This approach represents a more simple and safe method than other strategies that require endodermal differentiation because endoderm formation has already occurred by the time of isolation. Furthermore, selecting an endodermal source (e.g., intestine) with a close lineage relationship is logical since one is not trying to reprogram cells from ectoderm or mesoderm to endoderm (Wang et al., 2016). The transformation of digestive tract epithelial cells into endoderm cells using a small molecule cocktail has already been achieved and such cells are genetically stable (Wang et al., 2016). The strategy of using endodermal cells as initiators for differentiation can be less fraught, with greater chance of success and at far lower cost. This has been an increasingly interesting and promising strategy, but additional investigations are necessary to validate these early findings.

### 2.5 Hepatic Stem/Progenitor Cells

There are two types of multipotent cells in the liver: hepaoblasts and hepatic stem cells (HpSCs). Hepatoblasts are diploid bipotent cells with hepatocytes and cholangiocytes differentiation, locating in the canals of Hering in the adult liver. As the precursors of hepatoblasts, HpSCs are multipotent and can give rise to pancreatic islets cells except for hepatocytes and cholangiocytes. These cells can be found in the ductal plates of fetal and neonatal livers, and the canals of Hering in pediatric and adult livers. These two kinds of cells have very similar antigenic profiles, only with and without AFP expression, respectively (Chen et al., 2017; Schmelzer et al., 2007; Turner et al., 2011; Zhang, et al., 2008). These cells can be lineage-restricted into hepatocytes under different condition, which indicates that they are safe for use in transplantation *in vivo* (Cardinale et al., 2011; Turner et al., 2011). However, the extraction and separation of HpSCs or hepatoblasts presents a challenge due to the scant numbers of HpSCs (0.5–2.5% of liver parenchyma of all donor ages) and hepatoblasts (<0.01% in adult livers) (Turner et al., 2011; Liu et al., 2019). Although it is possible to obtain proliferative hepatoblasts by transferring both stem maintaining genes and liver specific genes, the final differentiation efficiency seems to be dissatisfactory (only 56.7%) (Yu et al., 2013; Park et al., 2019). Some scientists tried to change the composition of the culture medium and add some growth factors to transform mature hepatocytes into proliferative hepatoblasts, which have been realized in both mouse and human cells (Katsuda et al., 2017; Wu et al., 2017; Fu et al., 2019; Katsuda et al., 2019; Katsuda and Ochiya, 2019). Such chemically-induced hepatoblasts can stably expand *in vitro* and differentiate into mature hepatocytes under appropriate conditions and without gene mutations (Katsuda et al., 2017). The degree of differentiation of initial cells is close to the terminal state, and the inertia of cells makes it less steps to differentiate into HLCs.

## 3 Induction and Maturation of Hepatocyte-Like Cells *in vitro*

Reviewing the development history of the whole liver, it is not difficult to find that process is committed and complex. Immature stem cells develop into polarity and functional maturation hepatocytes, initiated by exogenous signals, cell localization clues and accumulated transcription factors, which is inseparable from the transduction and regulation of chemical and mechanical signals (Trefts et al., 2017; Ober and Lemaigre, 2018). Therefore, in the induction of HLCs *in vitro*, from the initial attempt to stimulate cell differentiation by adding a certain proportion of xenobiotics, in recent years, increasing researches also take into account the interaction between cells and cells and the extracellular matrix. The development from monolayer to multilayer differentiation and even multicellular culture has promoted the maturation of HLCs *in vitro* significantly (Kaserman and Wilson, 2017; Tomizawa et al., 2017; Blau and Miki, 2019; Mun et al., 2019).

### 3.1 Chemical Approach, Adding Exogenous Substances

**FIGURE 3:**
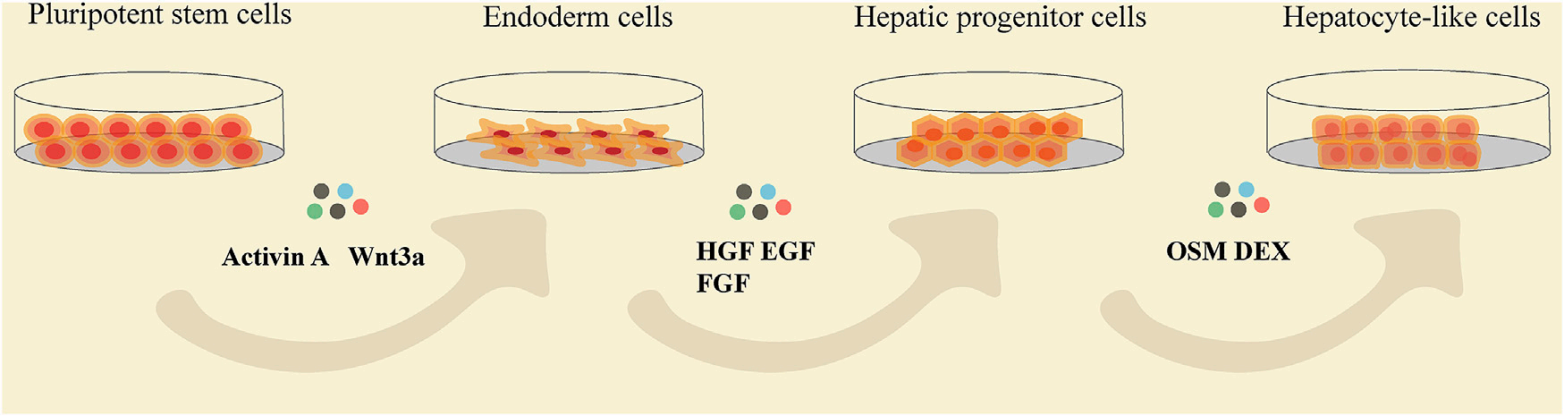
The flow chat showing the stages of pluripotent stem cells differentiating into HLCs and the common cytokines added at each differentiation stage. HGF, hepatocyte growth factor; EGF, epidermal growth factor; FGF, fibroblast growth factor; OSM, oncostatin M; DEX, dexamethasone.

Using different proportions of cytokines and growth factors based on activation or inhibition of signals on a regular basis is the basic induction method of generating HLCs *in vitro* (Figure 3). Generally, totipotent cells need to go through three stages to differentiate into HLCs in natural state (Table 1). Activin A acts via BMP signaling pathway, which often is coupled with Wnt3a during the highly efficient induction of definitive endoderm from pluripotent stem cells (Hay et al., 2008; Mitani et al., 2017; Si-Tayeb et al., 2010). And this process is considered to be the premise of formation of available HLCs *in vitro*. Hepatic nuclear factor (HGF), epidermal growth factor (EGF), FGF, and other growth factors are commonly used, which mainly promote the differentiation of endodermal cells into hepatocytes and inhibit the differentiation of non-hepatocyte cells (Raoufil et al., 2015; Vojdani et al., 2015; Kaserman and Wilson, 2017). In the process of HLCs generation, the key is to promote and induce the mature phenotype of cells, which determines the authenticity and safety of the experiments based on it. Generally, dexamethasone (DEX), oncostatin M (OSM) are often added at the mature stage to increase the expression of maturation HLCs genes and enhance their functions (Tomizawa et al., 2017). OSM is a key factor involved in the development and maturation of fetal liver, and OSM can also promote hepatic progenitor cells to hepatocyte maturation when adult liver injured (Kamiya et al., 1999; Okaya et al., 2005). *In vitro* culture, the addition of OSM combined with DEX which is prominent in inducing the expression of cytochrome enzyme in hepatocytes, can significantly increase hepatic protein synthesis was demonstrated (Lindley et al., 2002; Chivu et al., 2009; Zhang et al., 2012). The use of cytokines to induce hepatocyte formation is a classic method with high success rates, but this technique is often accompanied by the high costs and poor efficiency, and cannot meet clinical needs. Obviously, the induction of HLCs only with growth factors is no longer a routinely induction pathway because of its high cost and long time (about 15–28 days). However, this method is still as the basic idea to combined with other improved methods for yielding HLCs.

**TABLE 1:** Hepatocyte-like cells formation by cytokines and growth factors.

Cell source	Endoderm formation	Hepatic specification	Maturation	Days	Ref.
Foreskin fibroblast-derived iPSCs	100 ng/ml Activin A	1% DMSO	30 ng/ml OSM	19	Wang et al. (2016)
	50 ng/ml Wnt3a		50 ng/ml HGF		
			10 μmol DEX		
iPSCs	10 ng/ml BMP4	50 ng/ml BMP4	100 ng/ml HGF	25	Kaserman and Wilson (2017)
	10 ng/ml VEGF	10 ng/ml FGF2	20 ng/ml OSM		
	10 ng/ml FGF2	10 ng/ml VEGF	6 μmol Vk		
		10 ng/ml EGF	100 nmol DEX		
		20 ng/ml TGF-α			
		100 ng/ml HGF			
		100 nmol/L DEX			
iPSCs	100 ng/ml Activin A	20 ng/ml BMP4	20 ng/ml HGF	15	Kehtari et al. (2018)
		10 ng/ml FGF-2	20 ng/ml OSM		
			DEX		
ESCs	100 ng/ml Activin A	20 ng/ml BMP2	ITS	22	Kim et al. (2015)
		30 ng/ml FGF4	10 ng/ml OSM		
		2 μmol/L RA	DEX		
		10 nmol nicotinamide	20 ng/ml HGF		
		1 ng/ml b-FGF			
		100 μmol/L Vc			
ESCs/iPSCs	100 ng/ml Activin A	20 ng/ml BMP4	20 ng/ml OSM	20	Si-Tayeb et al. (2010)
		10 ng/ml FGF2			
		20 ng/ml HGF			

Cell source	Hepatic specification	Maturation	Days	Ref.
WJ-MSCs	10 ng/ml FGF4	20 ng/ml HGF	21	Vojdani et al. (2015)
	20 ng/ml HGF	20 ng/ml IGF		
	20 ng/ml IGF	100 nmol/L DEX		
	100 nmol/L DEX	10 ng/ml OSM		
UV-MSCs	500 nmol DEX	10 ng/ml EGF	28	Raoufil et al. (2015)
	1 × ITS	20 ng/ml b-FGF		
	50 ng/ml HGF	1 × ITS		
	10 ng/ml EGF	50 ng/ml OSM		
	20 ng/ml b-FGF			
AD-MSCs	20 ng/ml HGF	20 ng/ml HGF	21	Shabani Azandaryani et al. (2019)
	DEX	DEX		
	20 ng/ml IGF-I	20 ng/ml IGF-I		
	10 ng/ml OSM			

Abbreviation: FGF, 4, fibroblast growth factor 4; HGF, hepatocyte growth factor; IGF, insulin like growth factor; DEX, dexamethasone; OSM, oncostatin M; ITS, insulin/transferrin/selenium; EGF, epidermal growth factor; b-FGF, basic-fibroblast growth factor; DMSO, dimethyl sulfoxide; BMP4, bone morphogenetic protein 4; VEGF, vascular endothelial growth factor; TGF-α, transforming growth factor-α; Vk, vitamin K; RA, retinoic acid; Vc, ascorbic acid.

Small molecules, economic and effective substitutes for cytokines, can modulate gene expression and epigenetic modifications, accelerate the differentiation process, and promote maturation in hepatocytes (Du et al., 2018; (Qin et al., 2018; Tasnim et al., 2015) (Table 2). The chemical inhibitors GSK-3β, CHIR99021 and 6-bromo-indirubin-3′-oxime can activate Wnt signaling, regulate SOX17 expression, and promote dedifferentiation (Tasnim et al., 2015; Xu et al., 2015; Huang et al., 2017). The use of CHIR99021 reduce concentrations of activin A without affecting the differentiation rate of endoderm (Farzaneh et al., 2018). Sodium butyrate and valproic acid are histone deacetylation inhibitors that can promote the differentiation of definitive entoderm into liver-specific cells (Kondo et al., 2014; Panta et al., 2019). Trichostatin A, 5-aza, and nanomycin A, all of which are epigenetic modifiers, can be employed to induce differentiation of HLCs (Seeliger et al., 2013; Cipriano et al., 2017; Nakamae et al., 2018). FH1 and FPH1 have been used to replace HGF and OSM, respectively, to promote hepatocyte maturation. When used in conjunction with A83-01, dexamethasone, and hydrocortisone, the rate of cell differentiation was increased to 67.7% (37.1% in the cytokines cocktail group) (Shan et al., 2013; Du et al., 2018).

**TABLE 2:** Small molecules and possible mechanisms in HLCs formation.

Effect	Small molecules	Mechanism	Cell application	Ref.
Endoderm induction	IDE1	similar to activin A, induces Smad2 phosphorylation and drives AD-MSCs to endoderm formation	AD-MSCs	Xu et al. (2015)
	CHIR99021	a specific chemical inhibitor of GSK-3, can induce a rapid increase in the expression of the endoderm makers	AD-MSCs	Xu et al. (2015)
			ESCs	Siller et al. (2015)
			iPSCs	Du et al. (2018)
	6-bromo-indirubin-3′-oxime (BIO)	a GSK-3 inhibitor, mimics activation of Wnt signaling	ESCs	Tasnim et al. (2015)
	LY294002	inhibits maintenance of pluripotency and promotes differentiation to endoderm	ESCs	Tasnim et al. (2015)
Promotion of liver-specific induction and maturation	SJA710-6	a novel small molecule, can improve the process of hepatic differentiation by regulating the high expression of FOXH1 (FAST1/2)	MSCs	Ouyang et al. (2012)
	dimethyl sulfoxide (DMSO)	drives endoderm toward a hepatic fate and promotes maturation	ESCs	Tasnim et al. (2015)
			iPSCs	
			NMSCs	Cipriano et al. (2017)
				Du et al. (2018)
				Siller et al. (2015)
	Ile-(6) aminohexanoic amide (Dihexa)	an HGF receptor agonist, can promote hepatic maturation	ESCs	Siller et al. (2015)
			iPSCs	
	sodium butyrate (SB)	a histone deacetylase inhibitor, results in high levels of hepatic marker expression and reduces cell death	ESCs	Tasnim et al. (2015)
			WJ-MSCs	Du et al. (2018)
				Panta et al. (2019)
	SB431542	a TGF-β inhibitor, is used for the differentiation of progenitors to HLCs	ESCs	Tasnim et al. (2015)
	5-Azacytidine (5-aza)	a DNA methyltransferase inhibitor, epigenetic changes support the hepatic differentiation	NMSCs	Cipriano et al. (2017)
	Trichostatin A	a histone deacetylase inhibitor, improves hepatocyte phenotype	NMSCs	Cipriano et al. (2017)
			AD-MSC	
	A83-01	a TGF-β inhibitor, is continuously used to promote hepatocyte differentiation	ESCs	Du et al. (2018)
			iPSCs	
	FH1 and FPH1	are used to replace HGF and OSM to promote hepatocyte generation	ESCs	Du et al. (2018)
			iPSCs	

Abbreviations: AD-MSC, adipose-derived mesenchymal stem cells; ESCs, embryonic stem cells; iPSCs, induced pluripotent stem cells; MSCs, mesenchymal stem cells; NMSCs, neonatal mesenchymal stromal cell; WJ-MSCs, Wharton’s Jelly-derived mesenchymal stem cells; GSK-3, glycogen synthase kinase 3; TGF-β, transforming growth factor-β; OSM, oncostatin M.

Much controversy exists regarding the use of dimethyl sulfoxide (DMSO); in particular, its dose may affect the differentiation results. Indeed, studies have shown that 0.1% DMSO can accelerate the morphological differentiation of stem cells, whereas 1% or 0.5% DMSO can enhance the differentiation of liver specificity (Siller et al., 2015; Alizadeh et al., 2016). Contrary to this conclusion, Wang et al. pointed out that the differentiation efficiency of cells was not affected with the use of DMSO (Wang et al., 2019). Nevertheless, as a sulfur-containing organic compound, DMSO can interact with protein hydrophobic groups, resulting in protein denaturation, affecting cell metabolism and free radical scavenging, which also contribute to its controversial use.

In brief, exogenous substances are added to simulate cytochemical signals and the paracrine mechanism of cell development *in vivo*. The application of classical cytokines to small molecules is not only an innovative, simplified induction method but also the embodiment of deep insight into cell development and differentiation. Although the induction method involving small molecules is simple and cost-effective and can even be used to replace growth factors, screening an effective small molecule is a time- and money-intensive process (Siller et al., 2015). Generally, a mixture of growth factors and small molecules has been shown to effectively induce directional hepatic differentiation from stem cells. However, this approach still suffers from challenges in finding the most appropriate mixture proportion once the culture system becomes complex, such as the need to regulate the fate of different cell types at the same time.

### 3.2 Culture System

It is also important to provide appropriate mechanical stimulation and growth space for cells to further promote differentiation and maturation. Therefore, a number of studies have sought to change the physical environment of cell growth, including the matrix, oxygen concentration, and flow effect, using different culture systems.

### 3.2.1 Spheroid Culture

Spheroid cultures involve the self-aggregation of cells in static culture systems, such as low-adhesion culture plates or suspension cultures, resulting in the formation of cell spheres. The spatial distribution formed by the cells in the sphere is conducive to the extension of the three-dimensional (3D) structure of the cells that can show the function of the cells better. Meanwhile, the cells separated from the single mode of monolayer growth in the dish can be more beneficial for absorption and exchange of nutrients and promote the maturation of HLCs *in vitro* (Lauschke et al., 2016; Choi et al., 2018). Studies have shown that HLCs in spheroid culture show increased expression levels of liver-specific genes, cytochrome enzymes, and esterase, with the appearance of bile canaliculi (Kim et al., 2015; Choi et al., 2018). However, the size of the aggregates formed by HLCs affects nutrient exchange and signal reception by cells in the sphere, thus affecting their subsequent differentiation (Meier et al., 2017). Extremely small spheroids result in the loss of cells if there is fluid shear stress in the culture. Large-sized spheroids may exhibit issues regarding the diffusion of oxygen and metabolism of substances in cells within the spheroids, resulting in inconsistent differentiation of the whole spheroid (Farzaneh et al., 2018). Recently, Zeinab et al. placed particles containing growth factors in the center of such spheres to ensure an evenly distributed release of growth factors, thereby reducing the otherwise uneven absorption of nutrients at the center of the sphere (Heidariyan et al., 2018).

### 3.2.2 Organoid Culture

Organoid cultures, comprising parenchymal cells along with one (or more) mesenchymal cell types, reproduce primary tissues more accurately and incorporate more of the original developmental processes of cells (Koike et al., 2019). Compared with spheroid cultures, liver organoids are superior in terms of cell diversity and long-term culture *in vitro* (Mun et al., 2019). The effective microvascular structure formed by organoids can provide oxygen and nutrients to the cells in the center, thus improving the maturity of organoids and prolonging the culture time *in vitro*. Organoids with microstructures and microvasculature show irreplaceable advantages in recapitulating organogenesis and as the alternative treatment for organ failure (Takebe et al., 2017; Koike et al., 2019). At present, the use of organoids is the most widespread method to implant cells in Matrigel domes along with different cytokines to promote differentiation. However, the need for an extracellular matrix (e.g., Matrigel) to maintain long-term culture introduces undefined components, making it difficult to reproduce appropriate culture conditions. In addition, the size of organoids formed by this method is limited, and it is still a simplified organ compared to the native tissue with complex architecture and cellular diversity (Brassard et al., 2021). Although the continuous constructional improvements of organoid platforms are gaining momentum and result in improved physiological interactions between different systems (e.g., immune system and vascular system), cultivating multiple cell types on a single platform is still a challenge. Furthermore, effective replication of the cellular diversity of the liver is a time- and money-consuming process (Harrison SP et al., 2021; Shiota et al., 2021).

### 3.2.3 Culture Based on Hydrogel

Hydrogels are a type of polymer that can swell in water, providing an extracellular matrix by coating culture dishes, there by simulating the physiological growth of cells and promoting the diffusion of nutrients and cellular growth factors. As a biomaterial, hydrogels also play a non-negligible role in regulating cell proliferation, activity, and differentiation when they become part of the microenvironment of culture systems (Lutolf and Hubbell, 2005; Biggs et al., 2010). Hydrogels possess beneficial inherent chemical properties, as well as ideal wettability, roughness, and stiffness, which may affect cell growth, adhesion, migration, and apoptosis (Gentile et al., 2010; Slepicka et al., 2015). Hydrogels are classified into natural and synthetic polymer hydrogels, depending on their source. Natural hydrogels include alginate, collagen, and gelatin, while synthetic polymers include polyacrylamide (Toivonen et al., 2016; Luo et al., 2018; Ma and Huang, 2020).

A natural hydrogel derived from the decellularized extracellular matrix (ECM) of animal liver tissue not only provides a complex scaffold structure but also preserves the active substances present in it, including collagen, fibronectin, and glycosaminoglycans, as well as HGF, bFGF and other growth factors, in the cell growth microenvironment (Wang et al., 2016; Lorvellec et al., 2017; Wang et al., 2018). Spheroids formed by stem cells in liver ECM hydrogels have a smooth surface and are homogeneous in size (Toivonen et al., 2016). This method promotes the expression of maturation genes, such as *ALB* and *CYP3A4*, in HLCs, while effectively reducing the expression of *AFP* (Wang et al., 2016). However, polypeptides in natural hydrogels (e.g., collagen type I) contain animal-sourced antigens; this characteristic reduces their biological safety and thus limits their clinical applications. Recyclable mixed hydrogels with stable chemical properties and the plant-derived biomaterials known as cellulose nanofibrils, which are nontoxic, biocompatible, and biodegradable, have gained attention recently (Chitrangi et al., 2017; Poorna et al., 2021).

Polymeric synthetic hydrogels have similar structures and properties to natural ECM, providing suitable mechanical simulation and adhesion sites for the formation and maturation of HLCs (Yamazoe et al., 2013; Mahmoodinia Maymand et al., 2017). Common scaffold materials include poly L-lactic acid, polyether sulfone, and polycaprolactone. Because of the difference in the synthesis process and material source, cell adhesion, growth, and differentiation are affected (Biggs et al., 2010). Thus, aligned polyethersulfone synthesized by electrospinning technology is more conducive to the differentiation of HLCs and increases the expression of CYPs than random polyethersulfone (Mahmoodinia Maymand et al., 2018); this difference may be attributed to the fact that orderly arrangement of materials is beneficial for the formation of highly ordered tissue assemblies. Furthermore, compared with single polymers, hybrid scaffolds have better biocompatibility and material properties and can effectively improve the phenotype of HLCs and maintain phenotype stability *in vitro* (Mahmoodinia Maymand et al., 2017; Mobarra et al., 2019). For example, mixed scaffolds comprising poly L-lactic acid and collagen-I have a clear fiber structure, which can improve the maturation of hepatocytes and simplify the differentiation process (Wang et al., 2016).

Hydrogels can be used for single-layer cultures or covered with the same or different matrices to form a so-called sandwich culture (Bi et al., 2006). Sandwich culture promotes cell growth by maintaining material exchange on top of the substrate and a stable cell culture *in vitro,* providing an effective hepatotoxicity prediction model (Bi et al., 2006; Sakai et al., 2019). Unfortunately, this method is limited by the inability to remove apoptotic cells. Furthermore, an obvious shortcoming is that the extract of proteins from cells always mixes with exogenous proteins present in polypeptide-based hydrogels, leading to experimental difficulties.

### 3.2.4 Hydrogels in 3D Bioprinting

The controllable viscosity and water storage ability of hydrogels, as well as excellent cytocompatibility, make then the ideal choice for 3D bioprinting technology (Irvine and Venkatraman, 2016). Bio-inks composed of hydrogels and cells allow the replication of functional organs with tissue structure during printing; moreover, the location of cells can be preset in order to simulate natural tissues more accurately and show better therapeutic effects in disease treatment (Wust et al., 2011; Brassard et al., 2021). It is expected that HLCs are more mature both in liver phenotype and function after incubation on 3D-printing scaffolds (Kang et al., 2018). As a vital part of 3D printing, hydrogels can not only provide temporary residence for the isolated cells but also stabilize cells in the printing process to avoid thermal and mechanical damage and ensure the survival rate of cells (Belk et al., 2020). Indeed, potential pollution in the process of *in vitro* printing and the product damage owing to toxic particles produced by the materials, cannot be ignored. Notably, the effects of material factors on cell differentiation should also be controlled.

### 3.2.5 Bioreactor

Bioreactors can comprehensively simulate microenvironments suitable for hepatocyte growth *in vivo* and enable scaling-up of the cell culture system (Ardalani et al., 2019; Yamashita, et al., 2018). The bioreactor is equipped with parameter setting systems, which can realize the real-time monitoring and adjustment of temperature, oxygen concentration and shear force in the incubator. By enabling fluid flow in the culture medium, simulating the flow in peripheral blood vessels experienced by hepatocytes *in vivo*, the cells are always exposed to consistent concentrations of nutrients and oxygen (Yen et al., 2016; Kehtari et al., 2018). Such dynamic culture systems can remove unhealthy cells with weak adhesion and dispose of cellular metabolites. For example, microfluidic-based biochips provide cells with a stable fluid-flow environment (Jang et al., 2019). The presence of a flow effect is expected to not only to improve the maturation of HLCs, but also to increase the levels of CYP1A2 activity. Furthermore, the effect of two-sided flow on cells is greater than that of one-sided flow set-ups. HLCs express increased levels of phase I and II enzymes, as well as undergo bile duct formation (Jang et al., 2019). Compared with static cell culture, ESCs cultured in stirred bioreactors can function as more mature HLCs, exhibiting upregulated liver gene mRNA transcripts and enhanced liver functionality (Park et al., 2014). In addition, bioreactors can maintain a relatively constant oxygen concentration in long-term culture. Research has demonstrated that the concentration of oxygen around cells can have a large impact on the state of cells (van Wenum et al., 2018; Kimura et al., 2019). IPSCs cultured under high oxygen levels differentiate into definitive endoderm more efficiently, and the expression of albumin and cytochrome enzymes in HLCs is significantly improved (Kimura et al., 2019). High oxygen (40%) conditions also promote the maturation of HLCs (van Wenum et al., 2018). However, Zhi found that the effects of hypoxia on liver differentiation depend on the duration of treatment, because short-term (24 h) hypoxic (10% O_2_) pretreatment can also increase hepatic gene expression and glycogen storage (Zhi et al., 2018).

Bioreactors enable simultaneous co-culture of various cell types. It is well known that nonparenchymal liver cells, such as endothelial sinus, Kupffer, hepatic stellate, and bile duct cells, play important roles in the process of liver development by secreting cytokines or contacting hepatocytes directly (Kitade et al., 2016). Co-culture with non-liver cells can prolong the culture time of hepatocytes *in vitro* and maintain the function of HLCs when cultured together with MSCs (Rebelo et al., 2017). MSCs not only provide signal transduction for HLCs, but also protect the spheroid from shear stress.

Microbioreactor represented by microfluidic biochips require low cost but high precision; therefore, they are usually used for high-throughput drug screening but not for large-scale cell preparation. Large bioreactors can increase cell production, especially when producing clinical quantities of cells (Tandon et al., 2013; Samal et al., 2019). However, because cells adhere to capillaries filled with nutrients and oxygen, the rate of perfusion and the properties of substances affect the efficiency of cellular metabolite exchange (Meier et al., 2017). Therefore, adjusting parameter variation to achieve the ideal differentiation effect *in vitro* has become one of the challenges in the popularization and application of bioreactors. Nevertheless, the use of bioreactors is still anticipated to become widespread owing to the quantitative advantage of cell culture.

### 3.3 Blastocyst Complementation

Although, to some extent, *in vitro* differentiation has been mimicking all the induction cues required for liver development *in vivo*, the immature and complex production processes are incompatible, resulting in a lag in clinical transplantation applications. Differentiation is optimally induced *in vivo*, where the host can provide all factors and conditions for cell development. Therefore, blastocyst complementation technology is used to confer a vacant developmental niche in the host via gene knockout, so as to provide a suitable growth environment for stem cells. Finally, the stem cells can compensate for the developmental vacancies and produce derived organs from donor cells (Wu et al., 2017; De Los Angeles et al., 2018; Crane et al., 2019). Using this strategy, human organs such as the pancreas, kidney, skeletal muscle, and liver, have been successfully derived from rodents and large non-rodents (Goncalves et al., 2008; Kobayashi et al., 2010; Usui et al., 2012; Matsunari et al., 2013). Recently, it was found that in the animal model of liver development disorder caused by deletion of the *HHEX* gene, normal liver could develop after blastomere supplementation in the embryonic stage; this result suggests that patient-derived iPSCs can be used to derive the mature liver tissue in some large animals suitable for *in vivo* transplantation (Matsunari et al., 2020).

### 3.4 Genetic Manipulation

Changing the expression of specific genes and introducing exogenous ones represent direct and effective strategies to regulate the function of differentiated HLCs *in vitro* (Table 3). The specific expression of target genes in host cells can be realized through a virus delivery system. For example, overexpression of liver-enriched transcription factors (*HNF4α* and *HNF1α*) and forkhead box (*FOXa2* and *FOXa3*) was found to shorten stem cell differentiation time, improve differentiation efficiency, and promote HLC maturation (Takayama et al., 2012; Hu et al., 2016; Hanawa et al., 2017). In addition, the use of adenovirus as a vector to transduce *ATF5*, *c/EBPα*, and *Prox1*, the three important mature hepatocyte transcription factors, into HLCs induced by traditional growth factors for 25 days, led to upregulated expression levels of liver markers, such as drug-metabolizing enzymes and liver cell metabolite transporters (Nakamori et al., 2016). Indeed, this genomic non-integration method has advantages for adjusting the poor metabolic function of HLCs, because it exhibits high transfection efficiency without the risk of insertion mutation.

**TABLE 3:** Application of gene editing technology in human HLCs formation.

Method of modification	Aim	HLCs generation (%)	Advantages	Limits	Example of cell types	Ref.
Lentivirus	overexpression of *HNF4α*	∼28%	induces HLCs directly and saves time and materials	genomic integration	immortalized BM-MSCs	Hu et al. (2016)
				poor transfection efficiency		
	overexpression of *HNF4α-1D*	N.D.	promotes definitive endoderm differentiation	genomic integration	iPSCs	Hanawa et al. (2017)
				poor transfection efficiency		
	overexpression of *FOXa3, HNF1a,* and *HNF4a*	∼20%	shows the function of mature hepatocytes	genomic integration	HFF1	Huang et al. (2014)
				proliferation arrest		
	overexpression of *FOXa3, HNF1a, HNF4a, ATF5, PROX1,* and *c/EBPα*	∼90%	generates functional HLCs efficiently and reproducibly	genomic integration	HEFs	Du et al. (2014)
				poor transfection efficiency		
	overexpression of *FOXa3, HNF1a,* and *GATA4*	N.D.	a non-invasive way as seed cells for reprogramming	genomic integration	UCs	Wu et al. (2020)
				poor transfection efficiency		
Adenovirus	overexpression of *FOXa2* and *HNF1α*	N.D.	promotes definitive endoderm differentiation and improves functionality of HLCs	instability of transgene expression	iPSCs and ESCs	Takayama et al. (2012)
	overexpression of *ATF5, c/EBPα,* and *PROX1*	N.D.	enhances the hepatic functions of HLCs	instability of transgene expression	iPSCs	Nakamori et al. (2016)
Transfect microRNA mimics	overexpression of miR-122, miR148a, miR-424, miR-542-5p and miR-1246	N.D.	induces HLCs directly and saves time and materials	long-term effect undefined	UC-MSCs	Zhou et al. (2017)
Electroporation	overexpression of miR-106a, miR-574-3p and miR-45	N.D.	induces HLCs directly and save times and materials	cell damage	UC-MSCs	Khosravi et al. (2018)
CRISPR/Cas9 system	PXR-mCherry	N.D.	can be used for identifying factors that increase PXR-mediated drug metabolism and hepatocyte proliferation	hard technique	iPSCs	Kim et al. (2018)
	target to *CYP3A4* locus	N.D.	realizes enrichment of high-functioning human iPSC-derived HLCs	hard technique	iPSCs	Takayama et al. (2018)

Abbreviations: BM-MSCs, bone marrow-derived mesenchymal stem cells; ESCs, embryonic stem cells; iPSCs, induced pluripotent stem cells; UC-MSCs, umbilical cord-derived mesenchymal stem cells; HEFs, human embryonic fibroblasts; HFF1, human fetal limb fibroblasts, UCs, urinary epithelial cells; N.D., no data.

Another approach is represented in the hepatic direct reprogramming, that is, human somatic cells bypass the induced pluripotent stage and directly reprogram into functional HLCs (Du et al., 2014; Huang et al., 2014; Wu et al., 2020) (Table 3). At present, induced functional HLCs have been successfully generated, by introducing a combination of some liver fate determined factors (e.g., *FOXa3, HNF1a, HNF4a,* and *GATA4*), from fibroblasts and urine epithelial cells (Huang et al., 2014; Wu et al., 2020). In addition, for further promoting the maturation of HLCs and their application in drug development, the overexpression of maturation factors (*ATF5, PROX1,* and *c/EBPα*) can greatly improve the level of drug metabolism enzymes, even comparable to human hepatocytes (Du et al., 2014). Although this approach is beneficial for the short-term induction of a large number of HLCs, it is known that liver development is a continuous change process, indicating that its network of expression regulation is continuous and complex (Ober and Lemaigre, 2018). Therefore, it is difficult to prove whether the constant expression of some genes can truly represent the differentiation of hepatocytes.

MicroRNAs (miRNAs), which regulate gene expression at the post-transcriptional level during cell development and growth, play significant roles during HLC induction. Zhou et al. found that a combination of five miRNAs (miR-122, miR148a, miR-424, miR-542-5p, and miR-1246) in cord mesenchymal stem cells could induce functional hepatocytes within 7 days without the addition of cytokines, providing a new strategy for *in vitro* induction of HLCs (Zhou et al., 2017). Among them, mir-122, as a liver-specific miRNA, exhibits the highest expression in the adult liver, accounting for approximately 70% of all cloned miRNAs. It plays an important role in the regulation of liver function and pathological development (Girard et al., 2008; Hu et al., 2012). Studies have shown that miR-122 can stimulate the expression of hepatocyte-specific genes and most hepatocyte-enriched transcription factors to form a positive feedback loop and induce hepatocyte differentiation *in vitro* (Laudadio et al., 2012). Overexpression of miR-106a, miR-574-3p, and miR-451 in cells resulted in formation of HLCs in 28 days; these HLCs expressed higher levels of ALB, cytokeratin (CK18), and HNF4α compared with cells induced by traditional cytokines (Khosravi et al., 2018). Moreover, the overexpression of miR-382 in rat hepatocyte progenitor cells promoted the maturation of HLCs (Zheng et al., 2018). In addition, miRNA induction methods usually require a combination of a variety of miRNAs; however, the network of miRNAs regulating gene expression is very complex. This characteristic increases the cost of the experiment because of the need for constant testing of new combinations to find an ideal one.

Overexpression of certain maturation genes in immature HLCs can optimize their liver-specific functions. Studies have shown that iPSC genome editing can be used to improve the expression of cytochrome enzymes and obtain high-purity CYP3A4-like hepatocytes that are needed to evaluate the risks of candidate drugs (Takayama et al., 2018). The CRISPR/Cas9 system has been used to establish a hepatocyte line with high *PXR* expression, which could promote the expression of iPSC-derived hepatocyte cytochrome enzymes and enhance cell proliferation capacity (Kim et al., 2018).

As mentioned earlier, compared with the traditional method of inducing cells from an undifferentiated state into appointed cells step by step, direct transdifferentiation based on genetic operation and epigenetic regulation has a higher efficiency and shorter cycle. However, the expression instability and tumorigenicity caused by the inherent defects of virus transfection may lead to inconsistent differentiation, which manifests itself in the mixed expression of immature hepatocyte progenitor cells and mature hepatocytes (Orge et al., 2020). While it is easy to induce epithelial stromal transformation in long-term *in vitro* culture, phenotypic instability can lead to poor transplantation outcomes *in vivo* (Xue et al., 2016). Although this drawback may lead to the limited application of this method *in vivo*, its application in drug screening and disease modeling cannot be ignored.

## 4 Applications and Challenges

### 4.1 Pharmaceutical Industry

**FIGURE 4:**
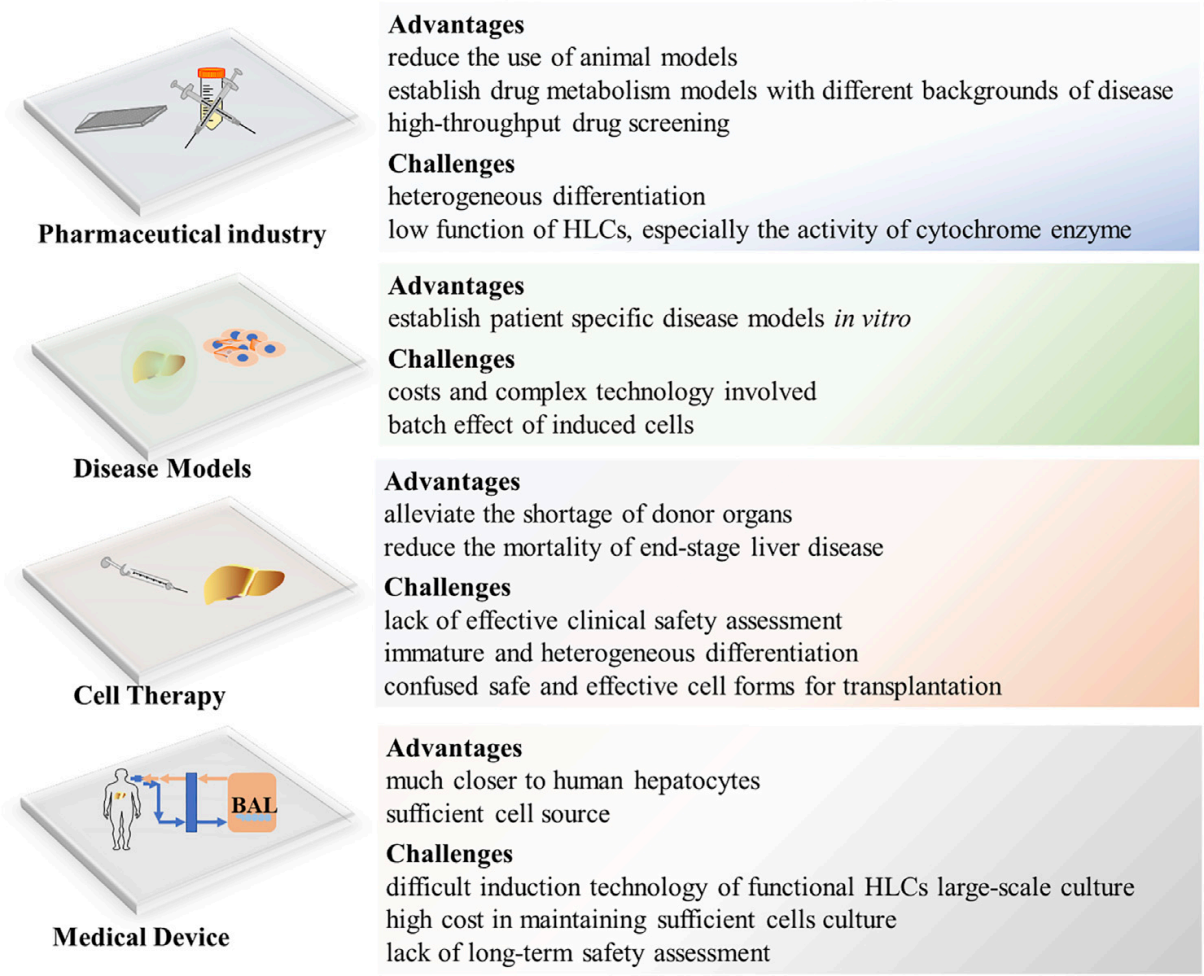
Applicaton and Challenges of HLCs, BAL, bioartificial liver.

Monolayer HLCs and organoid-derived HLCs provide high-throughput predictive models for drug screening and toxicity prediction and can become important drug research tools (Cayo et al., 2017; Shinozawa et al., 2021). In particular, HLCs in 3D culture show higher cytochrome enzyme activity and sensitivity to hepatotoxicants than those in 2D culture, as well as provide suitable platforms for drug screening (Lee et al., 2021). Meanwhile, special gene expression cell lines for scientific research can be established in combination with genetic manipulation technology. For example, *CYP2C19*-knockout human iPSC-derived HLCs can be used as a new CYP2C19-deficient metabolism model for drug research (Deguchi et al., 2019). The use of HLCs as a drug-screening model *in vitro* has been reported to be efficient, safe, and ethical (Williams, 2018). At present, the immature function of HLCs poses a significant challenge in toxicology studies. Although genetic editing can enhance the expression of some cytochrome enzymes, it is impossible to fully cover the CYP450 system containing all phase I and phase II enzymes. Therefore, HLCs cannot fully reproduce the oxidation–reduction reaction during drug metabolism (Figure 4).

### 4.2 Disease Models

Disease modeling from HLCs is not limited by the ethical issues of cell origin, because the current reprogramming technology of iPSCs can be applied to most adult cells in the human body, including the easily available urine epithelial cells and hair follicle epithelial cells (Zhou et al., 2011; Xu et al., 2018). In addition, HLCs can be used to establish disease models based on genetic backgrounds (e.g., autosomal recessive hypercholesterolemia) or specific disease models, such as *in vitro* HBV, HCV infection, and CYP2C19-deficient metabolism models (Schwartz et al., 2012; Sakurai et al., 2017; Deguchi et al., 2019; Nikasa et al., 2021). Additionally, expandable liver organoids provide a more favorable research tool for further exploring the etiology and pathophysiology of disease as a whole, not only from damaged cells but also from changes in the microenvironment (Gomez-Mariano et al., 2020; Ramli et al., 2020; Shinozawa et al., 2021). Considering that the occurrence and development of disease involve crosstalk and interaction between various cells and multiple systems, recent research has established a steatohepatitis model using multicellular cultured organoids, which presented a continuous pathological process of steatohepatitis from inflammation to fibrosis *in vitro* (Kisseleva and Brenner, 2019; Ouchi et al., 2019). As an *in vitro* research tool, organoids are not only highly physiologically related but also maintain genetic stability during long-term culture (Fiorotto et al., 2019). However, the costs and complex technology involved in establishing and maintaining organoid cultures are causes of the limited research. In addition, the batch effect caused by varying environments and culture durations affect the experimental results (Luce et al., 2021) (Figure 4).

### 4.3 Cell Therapy

HLCs are considered the most promising cells for liver regeneration and tissue engineering. Animal experiments have demonstrated that HLC transplantation in mice with liver injury significantly improves liver function and promotes liver regeneration (Park et al., 2019) and human iPSC combined with gene correction can induce normal hepatocytes to realize autologous cell therapy for patients with metabolic diseases (Yusa et al., 2011). Exploratory applications of HLCs in clinical treatments have shown satisfactory results (Mohamadnejad et al., 2010; Amer et al., 2011). Recently, liver organoid transplantation and cell sheet technology provide advanced methods to solve the loss in cell transplantation and improves therapeutic effects (Nagamoto et al., 2016; Tsuchida et al., 2020; Imashiro and Shimizu, 2021). However, cell therapy requires sufficient number of HLCs (2 × 10^8^/per injection) (Amer et al., 2011), which takes a certain time to extend such number of HLCs *in vitro*. But for patients with acute liver failure, 1 min less waiting will give them more chance to live. Therefore, it is very important to establish HLCs cell bank to store HLCs. Before that, we still need to solve the problems of low activity after long-term culture and cryopreservation of HLCs (Figure 4).

### 4.4 Medical Device

In bioartificial liver (BAL) research, there are mainly two cell lines employed; hepatoma cell lines and porcine hepatocytes, which have achieved ideal results. However, expandable PHHs may be more in accord with the characteristics of human liver metabolism and with ethical requirements. Compared with hepatoma cell lines, HLCs exhibit similarities to PHHs and show very substantial curative effects in treatment of a porcine acute liver failure (ALF) model (Shi et al., 2016). Recently, Li et al. developed a new BAL embedded with expandable liver progenitor-like cells from human primary hepatocytes for the treatment of an ALF porcine model, and the results showed that BAL attenuates liver damage, ameliorates inflammation, and enhances liver regeneration (Wei-Jian Li et al., 2020). Although stem cell-derived HLCs are considered the ideal cell source second to primary hepatocytes, their translation from laboratory to clinical application is limited by the difficult induction technology of functional HLCs large-scale culture and the high cost involved. In addition, whether BAL can be reused is still unclear because there is a lack of evaluation of the functional changes of HLCs before and after exposure to patient serum.

## 5 Conclusion

In conclusion, great progress has been made to improve the induction and culture of HLCs *in vitro* and enhance their potential applications. The increasing experiments suggest that cell fate is not only related to chemical signal, but also the mechanical signals and structural support provided by the extracellular environment are the key points to promote functional cells. Organ is a 3D architecture composed of cells, which means that co-culture of multiple cells and reasonable spatial distribution of cells are conducive to maturation of organ. Admittedly, that the maturity of HLCs has been improved to a certain extent, but the operation steps and culture system inevitably become complex, and there is no standard induction scheme to produce uniformly differentiated HLCs, which confuses the choice of induction protocol and rare replication of the same results. Although this review discussed fundamental and advanced methods in culturing HLCs, it inevitably puts too much focus on *ex vivo* research. Exploring the process of culturing functional hepatocytes *in vitro* will contribute to uncover the regulatory mechanism of cell fate and the interaction between microenvironment and cells, which is basic clues for disease modeling and personalized medicine. However, before the widespread application of HLCs in clinical treatment, there is still much research and investigation required, especially in terms of the safety of *in vivo* treatment.
